# Study of Microwave-Induced Ag Nanowire Welding for Soft Electrode Conductivity Enhancement

**DOI:** 10.3390/mi12060618

**Published:** 2021-05-27

**Authors:** Meng Zhang, Songjia Han, Zhi-Yang Xuan, Xiaohui Fang, Xiaoming Liu, Wu Zhang, Hui-Jiuan Chen

**Affiliations:** 1 Precision Medicine Institute, The First Affiliated Hospital of Sun Yat-Sen University, Sun Yat-Sen University, Guangzhou 510080, China; meng.zhang_china@outlook.com; 2 State Key Laboratory of Optoelectronic Materials and Technologies, School of Electronics and Information Technology, Sun Yat-Sen University, Guangzhou 510006, China; hansongjia@126.com (S.H.); ZYang_Xuan@outlook.com (Z.-Y.X.); 3 College of Physical and Material Engineering, Guangzhou University, Guangzhou 510006, China; fangxiaohui@gzhu.edu.cn; 4 College of Physics and Electronic Information, Anhui Normal University, Wuhu 241003, China

**Keywords:** Ag nanowires, microwave heating, thermal welding, glucose sensing

## Abstract

Silver nanowire (AgNW)-coated thin films are widely proposed for soft electronics application due to their good conductivity, transparency and flexibility. Here, we studied the microwave welding of AgNW-based soft electrodes for conductivity enhancement. The thermal effect of the microwave to AgNWs was analyzed by dispersing the nanowires in a nonpolar solution, the temperature of which was found to be proportional with the nanowire diameters. AgNWs were then coated on a thin film and welded under microwave heating, which achieved a film conductivity enhancement of as much as 79%. A microwave overheating of AgNWs, however, fused and broke the nanowires, which increased the film resistance significantly. A soft electrode was finally demonstrated using the microwave-welded AgNW thin film, and a 1.13 µA/mM sensitivity was obtained for glucose sensing. Above all, we analyzed the microwave thermal effect on AgNWs to provide a guidance to control the nanowire welding effect, which can be used for film conductivity enhancement and applied for soft and bio-compatible electrodes.

## 1. Introduction

Electrodes with good conductivity, conformability, and bio-compatibility are highly desired in soft electronics for signal sensing, motion detection and health care applications [1]. In early years, indium tin oxide (ITO) was used for soft electrodes due to its good transparency and conductivity. Nonetheless, ITO fabrication requires special material growth, high deposition temperature and vacuum processing [2]. In addition, ITO is also easy to crack when getting bent, which largely limits its applications in the fast-growing wearable electronic technology. Various other materials were later proposed to substitute ITO, such as conductive polymers, nanotubes and metallic nanowires, etc. [3,4,5,6,7,8]. However, polymer usually has the limitation of low conductivity, while the nanotubes could be undermined due to tube defects or bundling. Metallic nanowires (MNWs), instead, have the advantages of good flexibility, high transparency, large conductivity and cost effectiveness. Meanwhile, MNWs can be easily coated on soft thin films such as poly(ethylene terephthalate) (PET) or polydimethylsiloxane (PDMS), therefore they have become a promising candidate for soft electrodes in wearable technology [9,10,11,12].

When coated on thin films, the MNWs are physically contacted with each other and they form a network texture. The contact resistance between MNWs will inevitably affect the film electrical performance. Many remarkable works have been carried out to reduce the MNW contact resistance, including cold welding [6], plasmonic welding [13,14,15], laser sintering [16], mechanical pressure [17], thermal annealing [18,19], electron-beam-induced welding [20], solvent-induced welding [21,22] and so on. The above works can be generally categorized into pressure welding and heat welding approaches. The pressure welding approach loads large pressure on the whole device without selectivity, which is energy consuming and may lead to thin film damage. The traditional heat welding approach, instead, requires very delicate and expensive equipment to control thermal effect on a small selected area, which increases the cost and prevents large scale fabrication. Therefore, a simple welding method that satisfies both selective processing and large scale fabrication is highly demanded.

Microwave heating is a widely used method for the synthesis of nano-materials because of its advantages in thermal effect selectivity, reduced processing time and enhanced product purity [23,24]. Recently, some scientists proposed employing microwave treatment for MNW welding, which significantly improved the thermal and electrical conductivity of the nanowire-coated films [25]. While these preliminary works only focused on the demonstration of microwave welding of MNWs, a quantitative analysis of the microwave thermal effect on the MNWs is necessary for guiding the microwave welding control in real applications. In this work, we analyzed the microwave-induced temperature increase, welding and the conductivity enhancement of AgNWs at different nanowire densities, diameters and different microwave power and time applied. The conductivity enhancement was optimized for the AgNW-coated thin films, which was applied as a soft electrode for glucose concentration sensing.

## 2. Materials

Below are the materials for the AgNWs synthesis and experiment process: silver nitrate (AgNO_3_, CAS: 7761-88-8, 99.9999%, Sigma-Aldrich, St. Louis, MI, USA), cupric chloride (CuCl_2_, CAS: 7447-39-4, 99%, Sigma-Aldrich, St. Louis, MI, USA), ethylene glycol (EG, CAS: 107-21-1, anhydrous 99.8%, Sigma-Aldrich), polyvinyl pyrrolidone (PVP, CAS: 9003-39-8, MW = 360,000, Sigma-Aldrich), polydimethylsiloxane (PDMS, DC Sylgard 184), polyethylene terephthalate (PET, Dupont, Shenzhen, China). Before synthesis, AgNO_3_, CuCl_2_, and PVP were dissolved in EG to form a uniform solution.

## 3. Methods

### 3.1. Synthesis of AgNWs

The AgNWs were synthesized through the reduction of a metal salt precursor by a polyol. First, 20 mL ethylene glycol (EG) was heated to 155 °C to remove all water inside. Then, 160 µL (4 mM) CuCl_2_ solution and 6 mL (115 mM) polyvinyl pyrrolidone (PVP) solution were successively added into the EG with a 15 min interval time. Finally, 6 mL (95 mM) AgNO_3_ solution was added to the mixture, which was maintained at 155 °C for two hours to ensure a full synthesis of AgNWs. The AgNWs were then rinsed several times using acetone and deionized (DI) water for purification. The purified AgNWs were then dispersed in ethanol for next step experiments.

### 3.2. AgNWs Dispersion in Nonpolar Solution

**Figure 1:**
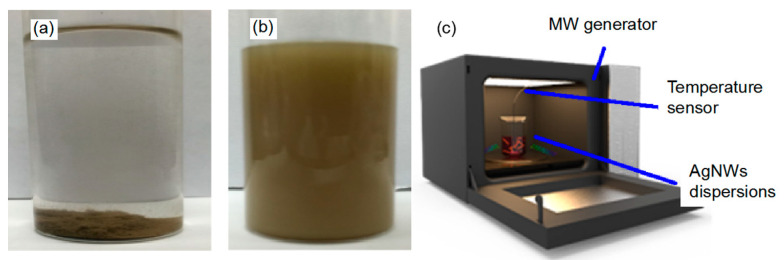
(**a**) AgNWs aggregation in Isopar H solution; (**b**) AgNWs dispersion in base solution of Isopar H, Span-20 and CH-5; (**c**) the setup for base solution heated by microwave.

AgNWs were dispersed into a base solution to evaluate the microwave thermal effect on the nanowires. Nonpolar liquid Isopar H was considered as the base solution, which has a low dielectric constant of 1.89 and high boiling point of 189 °C. Due to its non-polarity, Isopar H absorbs very little microwave energy and therefore will not affect the microwave heating analysis on AgNWs. AgNWs, however, are hydrophilic and will aggregate in Isopar H solution (Figure 1a). We therefore modified the base solution recipe by adding CH-5 hyperdispersant (5 wt%) and Span-20 surfactant (10 wt%) to the Isopar H. The AgNWs in ethanol from Section 3.1 were centrifuged at 5000 rpm for 5 min, and then heated at 70 °C for 10 min to remove the residual ethanol. AgNWs free of ethanol were quickly dumped into the base solution and agitated strongly with vortex mixer for 5 min. Homogenous AgNW dispersion was obtained, as shown Figure 1b. Microwave radiation was applied to the solution at a frequency of 2.45 GHz. The solution temperature was continuously monitored through a sensor system (OMEGA FOB-100 series) (Figure 1c) for quantitatively analyzing the microwave thermal effect on AgNWs.

### 3.3. AgNW Coated on PET Films

**Figure 2:**
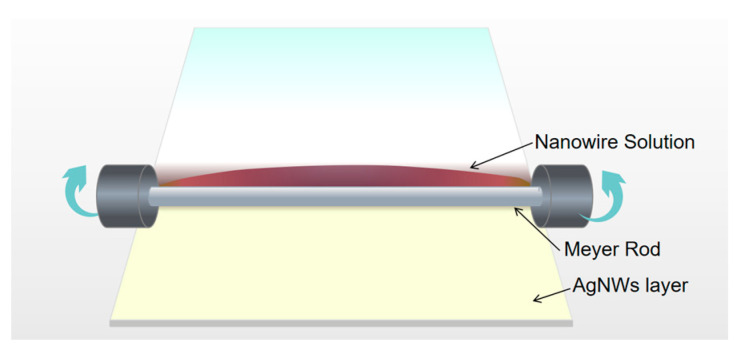
AgNW coated on PET films using Meyer rod.

The conductivity of AgNW-coated thin films can be enhanced by the microwave welding of nanowires. Here, a PET film was used as the nanowire substrate for its good transparency and softness, which is suitable for soft electronic applications. The synthesized nanowires were first dispersed uniformly in ethanol at weight concentration 0.0125 wt%, and then dropped along one edge of the PET film. A Meyer rod was used to roll the solutions across the film, as shown in Figure 2. Multiple layers of AgNWs can be obtained by repeating the above steps multiple times, while the rolling direction was switched to the orthogonal one after each step to ensure better uniformity. The coated film was heated at 70 °C for 1 min to remove the residue ethanol.

### 3.4. AgNWs Coated on PDMS for Electrode Application

**Figure 3:**
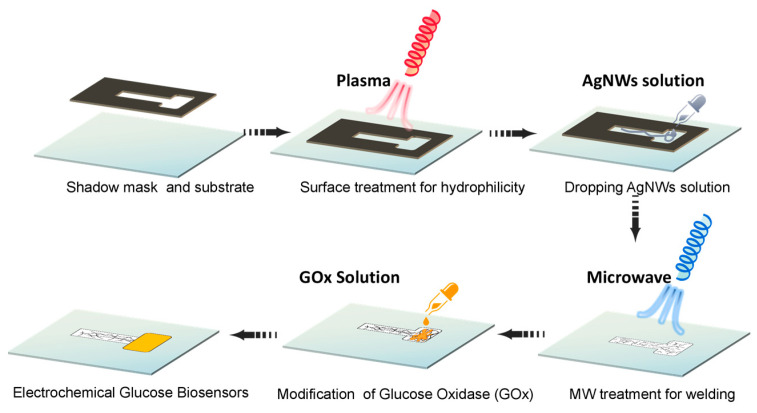
AgNW-coated PDMS electrode fabrication process.

We coated AgNWs on a PDMS film as a soft and bio-compatible electrode for biosensor application. The electrode was formed by first creating a hydrophilic area on the film using oxygen plasma treatment through a shadow mask, as shown in Figure 3. The AgNWs ethanol solution with nanowire diameter of 90 nm and concentration of 10 mg/mL was then dropped on the film. AgNWs adhered well on the hydrophilic area and formed the electrode pattern after the ethanol volatilized. The microwave was applied to the AgNWs for 30 s to improve the electrode conductivity. Glucose oxidase (GO_x_) was then dropped on the electrode for electrochemical sensing of glucose.

### 3.5. Cell Seeding for AgNW Bio-Compatibility Testing

We seeded T24 cells on four AgNW-coated PDMS thin films, which were then placed in a 48-well plate for AgNW bio-compatibility testing. On each film, about 20,000 cells in 100 µL completed medium were seeded, and then cultured in an incubator at 37 °C temperature and 5% CO_2_ concentration. The cell growth on the four culturing groups was investigated after 12, 24, 48 and 72 h, respectively. Meanwhile, the same amount of T24 cells were cultured directly in a fifth well for 72 h as a control group, while keeping other parameters the same. For cell growth investigation, 5 µM fluorescent dye of Calcein-AM (ThermoFisher C3100MP, Waltham, MA, USA) was used.

## 4. Results and Discussion

### 4.1. Structure Investigation of Synthesized AgNWs

**Figure 4:**
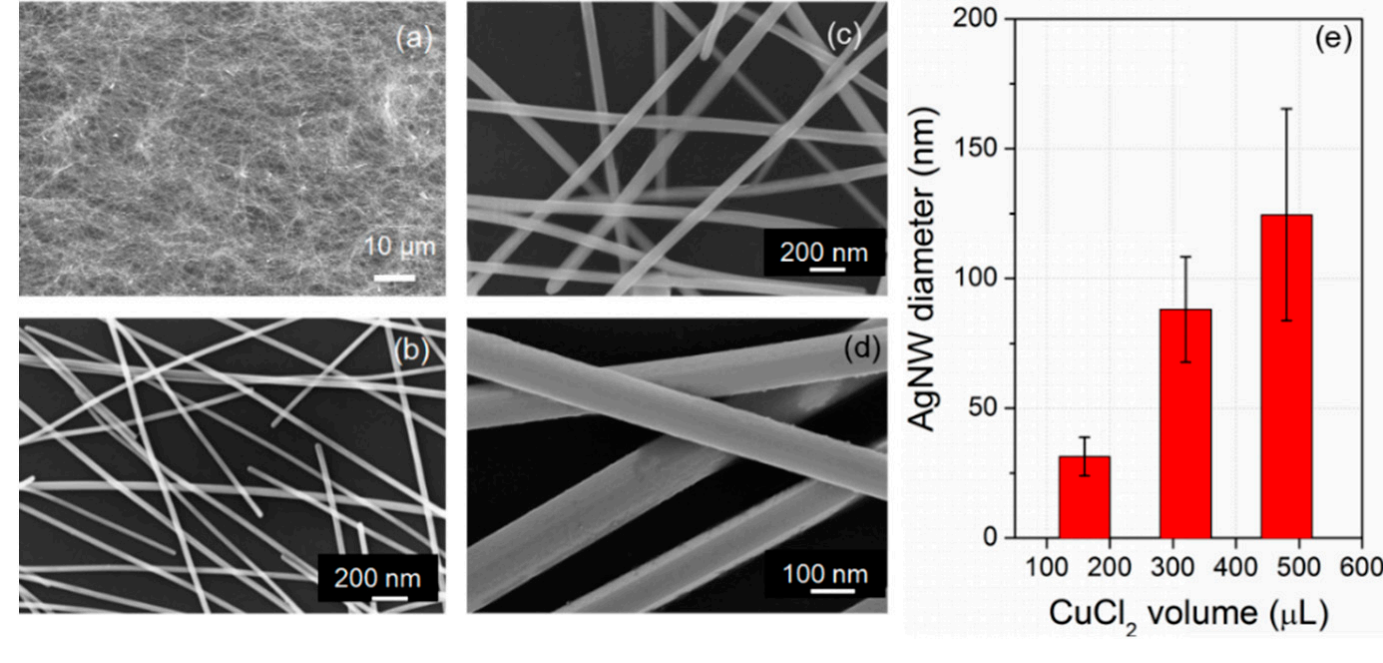
(**a**) AgNWs network; (**b**) close-up image of AgNWs with average diameter of 30 nm; (**c**) average diameter of 90 nm; and (**d**) average diameter of 120 nm. (**e**) The average diameter of AgNWs at different added CuCl_2_ volume in the process.

The synthesized AgNWs in Section 3.1 were coated on a thin PET film for structure investigation using an electronic scanning microscope (SEM, Hitachi S-4800) as shown in Figure 4a,b. The AgNWs formed a network structure on the thin film with a diameter if about 30 nm. The diameter of AgNWs can be adjusted by altering the amount of CuCl_2_ in the synthesis process. As shown in Figure 4c,d, the average diameter of AgNWs was increased to 90 nm and 120 nm when the added volume of the CuCl_2_ (4 mM) was increased to 320 µL and 480 µL in the synthesis process, respectively. Figure 4e shows the average diameters of the nanowires in different deviation values.

### 4.2. Microwave Heating on AgNWs in Nonpolar Solution

**Figure 5:**
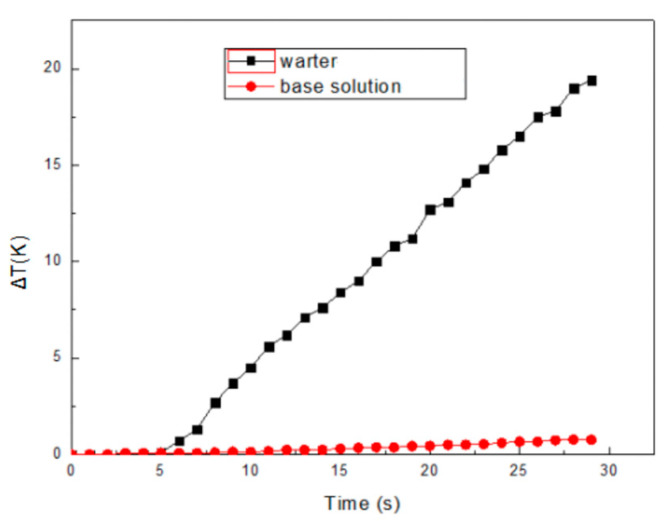
Temperature increase comparison for base solution and water under microwave heating.

To understand the microwave thermal effect on the AgNWs, we monitored the temperature of AgNWs dispersed nonpolar solution under microwave heating. Firstly, a 300 W microwave was applied to 20 mL Isopar H base solution (without AgNWs). For comparison, the temperature of the same volume of DI water under the same microwave radiation was measured. The initial temperature was set at room temperature, 25 °C. As shown in Figure 5, the temperature increase Δ*T*, was measured as low as 0.78 K for the base solution after a total 30 s time microwave heating, which, on the other hand, was almost 20 K for DI water. This confirmed that the base solution absorbs little microwave energy compared to conventional polar solvent.

**Figure 6:**
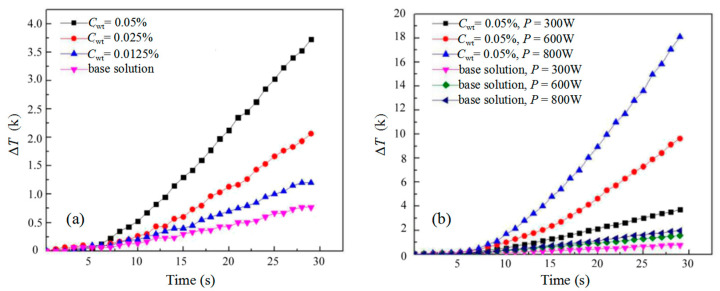
(**a**) Temperature change of AgNWs in the nonpolar base solution at various concentrations; (**b**) temperature change of AgNWs in the nonpolar base solution under different microwave powers.

We then monitored the temperature increase in the 20 mL base solution when dispersing different amounts of AgNWs in the solution. The initial temperature was kept at 25 °C. As microwave penetration depth of Ag (≈1.3 μm) [26] is much higher than the AgNW diameter, a strong electrical current in AgNWs is excited by the electrical and magnetic field of the microwave and a large amount of heat is generated [27]. Under 300 W microwave heating, the solution temperature increased by 1.22, 2.07 and 3.73 K after 30 s for the base solution with AgNW weight concentration *C*_wt_ = 0.0125%, 0.025% and 0.05%, respectively, as shown in Figure 6a. Considering the temperature increase in the base solution was 0.78 K, the additional temperature increase due to AgNWs was 0.44, 1.29 and 2.95 K for the above three concentration solutions, respectively. This indicated a proportional relation between the temperature increase and the AgNW weight concentration.

**Figure 7:**
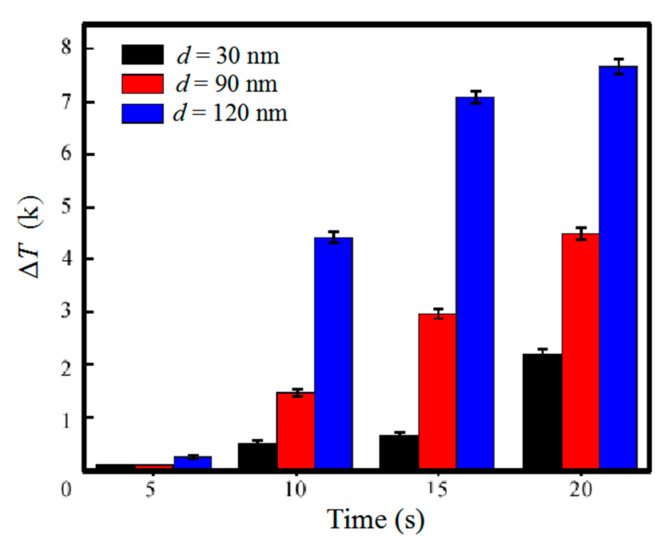
Temperature change of AgNWs dispersion at the power of 300 W with the concentration of 0.05 wt% for AgNWs with different diameters of 30, 90 and 120 nm.

To further analyze the microwave heating on AgNWs, we tested the thermal effect of 20 mL base solution without/with 0.05 wt% AgNWs under different microwave heating powers. After 30 s microwave heating, the temperature increased by 0.78, 1.54 and 1.99 K for solution without AgNWs, and by 3.73, 9.62 and 18.14 K for solution with AgNWs at microwave power of 300 W, 600 W and 800 W, respectively, as shown in Figure 6b. Therefore, the temperature increases due to AgNWs were 2.95, 8.08 and 16.15 K at microwave power of 300 W, 600 W and 800 W, respectively. It can be seen that the temperature change depended on the microwave power linearly. It is also worth noting that when the power increased from 300 W to 600 W and then to 800 W, the temperature of the base solution only increased by 97% and 155%, while the temperature increase due to AgNWs was 212% and 447%. This, again, demonstrated a high microwave power absorption ability of AgNWs. We then compared microwave heating on AgNWs of different diameters *d* = 30 nm, 90 nm and 120 nm in the 20 mL base solution at a fixed weight concentration of 0.05 wt%. The temperature increase of solution with different diameter AgNWs was recorded every 5 s, as shown in Figure 7, which was 2.3, 4.8 and 7.8 K for base solution with AgNWs in diameter of 30, 90 and 120 nm, respectively, indicating a linear relation of the microwave thermal effect with the nanowire diameter.

### 4.3. Microwave Heating of AgNWs on PET Thin Film

**Figure 8:**
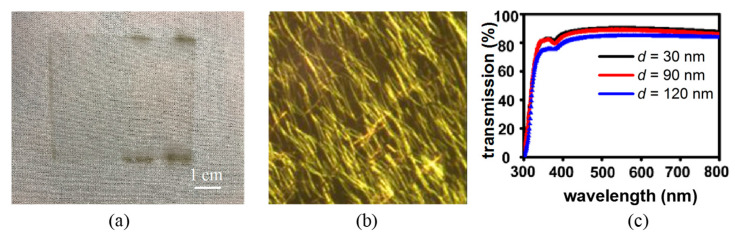
(**a**) Photograph of two layer AgNW-coated PET film; (**b**) nanowire structure under optical microscope; (**c**) optical transmittance of AgNWs film.

In this section we investigated the microwave welding of the AgNWs coated on a thin PET film, as shown in Figure 8a. The film was coated with two layers of AgNWs and manifested excellent transparency. A contacted network was observed under optical microscope, as shown in Figure 8b. The optical transmissions reached about 80% in the whole visible spectrum range for films with AgNW diameter of 30 nm, 90 nm and 120 nm, respectively, as shown in Figure 8c. The slight drop in the transmission for larger diameter AgNW film is resulted from the slightly higher light reflection by the larger AgNWs.

**Figure 9:**
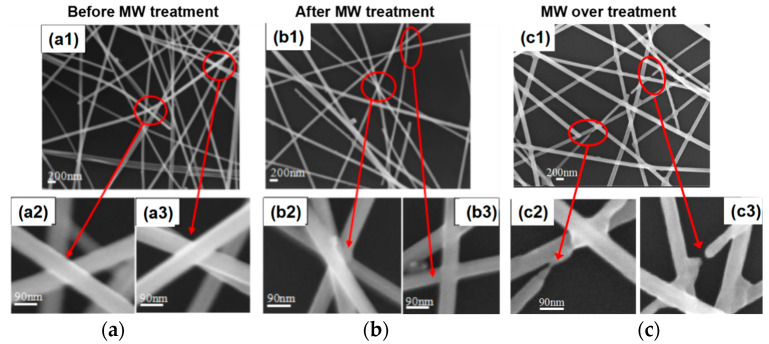
(**a**) AgNWs junction before microwave heating; (**b**) AgNWs junction after microwave heating; (**c**) AgNWs junction by an over-treatment of microwave.

The microwave welding effect of AgNWs on thin PET film was then evaluated using nanowires with diameter of 90 nm, which have a better transmittance than that of 120 nm AgNWs, and a stronger thermal effect than 30 nm AgNWs. The SEM image of the AgNW network is shown in Figure 9(a1) before microwave heating. The AgNWs randomly distributed on the film and contacted with each other as shown in Figure 9(a2,a3). The electrical resistance at the contacted junctions, namely contact resistance, was significantly higher than that of the nanowire itself, and could induce local hotspots by microwave heating [28]. After 30 s microwave heating, we observed that AgNWs started to weld together at some contact junctions (Figure 9(b3)), while remaining loosely contacted at the rest (Figure 9(b2)). This indicates that the heating was not uniform at all junctions. As the microwave heating continued to 90 s, most contact junctions were welded. In addition, the thermal effect was so large that some AgNWs started to melt (Figure 9(c2)) and even fuse, as shown in Figure 9(c3). The fused points were mostly closed to the contact junctions instead of at the contact junctions, implying that the heat generated was not enough to fuse welding junctions, but can only be conducted to nearby locations and fuse the nanowire.

**Figure 10:**
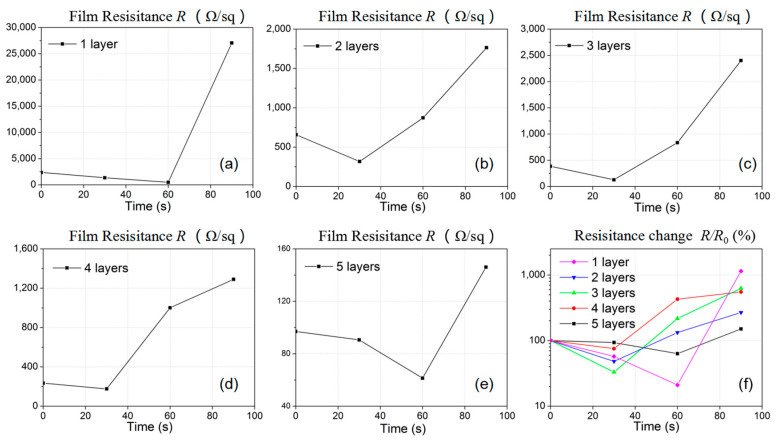
(**a**–**e**) Film resistance change with different time microwave heating with 1–5-layer AgNWs. (**f**) Relative resistance *R*_T_/*R*_0_ with different time microwave heating with 1–5-layer AgNWs.

The impact of microwave heating on the electrical resistance change of AgNW-coated thin film was investigated. The 90 nm diameter AgNWs of 1–5 layers were coated on five PET thin films, respectively, for resistance comparison. The thin film was heated by microwave radiation with power of 300 W at a frequency of 2.4 GHz, and the resistance changes were recorded every 30 s, as plotted in Figure 10. Before microwave treatment, the initial film resistance *R*_0_ was 2370, 658, 385, 234 and 97 Ω/m^2^ for films with 1, 2, 3, 4 and 5 layers of AgNWs, respectively, as indicated at the 0 s time in Figure 10a–e. In the first 30 s, the resistance started to decrease for all five thin film cases, which was due to the welding of the contacted junctions in the AgNW network and agreed with the SEM image as illustrated in Figure 9b. As the microwave irradiation time increased from 30 s to 60 s, the resistances for thin films with AgNWs of 1 layer and 5 layers continued to decrease due to the welding effect, while for thin films coated with 2, 3, and 4 layers of AgNWs, the resistance started to increase due to the fuse of the AgNWs, corresponding to the case in Figure 9c. After 60 s treatment, the nanowires were over heated by the microwave, and the nanowire fuse increased the electrical resistance significantly, which was even about 10 times larger than the original value for the 1 layer coated film under 90 s treatment. The relative electrical resistances *R*_T_/*R*_0_ for different AgNWs layered films are plotted in Figure 10f for comparison. It can be seen that the electrical resistance for films with 1 to 5 layers can be decreased by as much as 79%, 52%, 67%, 25% and 37% after the microwave treatment for 60, 30, 30, 30 and 60 s correspondingly. This indicates that the microwave welding time needs to be controlled to realize an optimized conductivity enhancement for the thin film.

### 4.4. AgNW Electrode for Glucose Concentration Sensing

**Figure 11:**
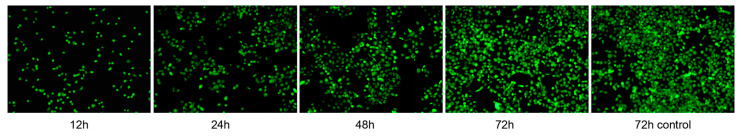
T24 cell growth on the AgNWs for 72 h, and comparison with that on the culture plate.

In this section, we coated the AgNWs on a thin film as working electrode for glucose concentration sensing application. We first tested the bio-compatibility of nanowire-coated PDMS film using T24 cells as described in Section 3.5. The cell growth conditions are shown in Figure 11. The cell number on the electrode grew stably and was found to be closed to that on the culture plate after 72 h growth. Therefore, the AgNWs are bio-compatible and suitable for the glucose concentration sensing.

**Figure 12:**
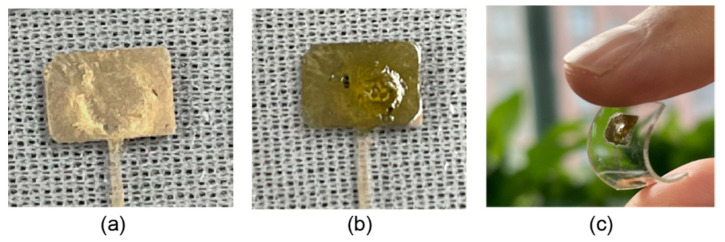
AgNWs electrode (**a**) before and (**b**) after GO_x_ coating; (**c**) the bending of the electrode.

**Figure 13:**
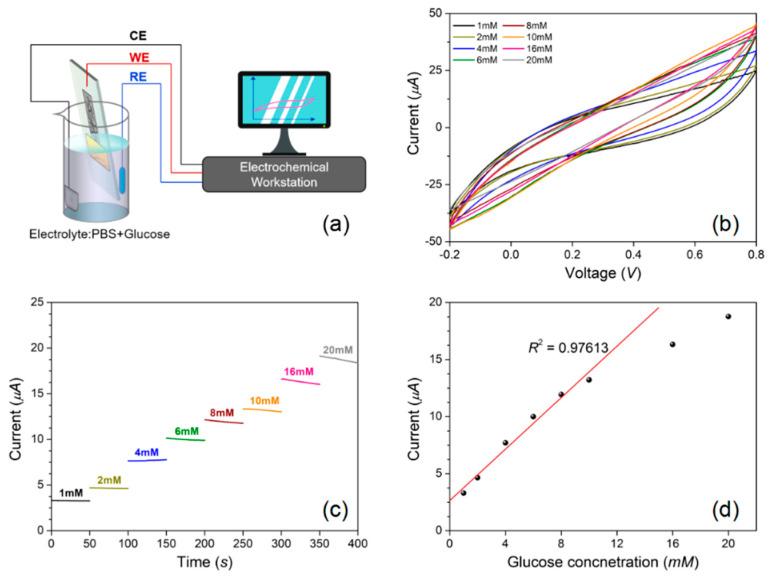
(**a**) Glucose sensing setup with the AgNW electrode; (**b**) cyclic voltammetry curve for the glucose sensing; (**c**) measured current for different glucose concentration; (**d**) average current at different glucose concentration and linear fitting.

Figure 12a,b shows the AgNW-coated PDMS electrode before and after coating the GO_x_. As shown in Figure 12c, this electrode is soft and can be bent flexibly, therefore it has significant advantages in conformal biosensing applications. The AgNWs film was then immersed into glucose containing PBS solution as the working electrode for concentration sensing, as shown in Figure 13a. An electrical potential *V*_glucose_ was applied between the working electrode and the counter electrode and the electric current from the working electrode to the reference electrode was measured. The cyclic voltammetry test was carried out by varying *V*_glucose_ between −0.2V and 0.8V, as shown in Figure 13b. It can be seen that the reduction peaks occurred at *V*_glucose_ = 0.6V in the cyclic voltammetry curve for different glucose concentrations. This voltage was set for the glucose concentration sensing. The glucose concentration was increased every 50 s, and the measured electrical current increased correspondingly, as shown in Figure 13c. The average current at each concentration was plotted in Figure 13d, and followed a good linear relation with the glucose concentration between 1 mM and 10 mM regime. The slope of 1.13 and *R*^2^ value of 0.976 were obtained through linear fitting analysis, indicating a sensitivity of 1.13 µA/mM in this concentration regime. The electrical current values at glucose concentration of 16 mM and 20 mM were far below the fitting line, indicating that the sensitivity began to decrease in this high concentration regime.

## 5. Conclusions

In summary, we investigated the thermal effect of a microwave on Ag nanowires, and realized a significant conductivity enhancement on AgNW-coated thin film due to microwave welding. A soft and bio-compatible electrode based on the nanowire coating and microwave welding were demonstrated and was applied for glucose concentration sensing. The microwave heating on AgNWs was investigated by dispersing the nanowires in a nonpolar base solution. Linear relationships were obtained between the solution temperature increase and the nanowire weight concentration in the solution, nanowire diameter and applied microwave power. Nanowire welding was observed at the contact junctions under microwave radiation, and an electrical resistance reduction of 79% was achieved. Finally, we demonstrated a good bio-compatibility of the AgNW-based soft electrode, which realized a 1.13 µA/mM sensitivity for glucose sensing application.

## Data Availability

Raw data presented in this study are available on request from the corresponding author.
